# Supplementary material for: Safety and effectiveness of stereotactic radiosurgery for larger hemangioblastomas (>2cc): A multi-center retrospective study
Source: PLoS One. 2026 Apr 17;21(4):e0337000. doi: 10.1371/journal.pone.0337000 (PMC13089703; doi:10.1371/journal.pone.0337000)
Supplement: Attachment — Submitted filename: PLOS ONE - SRS for larger hemangioblastomas.pdf [file pone.0337000.s001.pdf]

I was invited to review a paper titled "Safety and effectiveness of stereotactic radiosurgery for larger hemangioblastomas (>2cc): a multi-center retrospective study" for PLOS ONE. This is a large multi-center retrospective study evaluating SRS for larger hemangioblastomas (>2cc), comparing VHL-associated and sporadic cases. The topic is clinically relevant and addresses a gap in the literature, as most prior SRS studies have focused on smaller lesions. The dataset is sizeable, spanning multiple international centers, and the results are clearly presented.

Please find my comments below:

- The authors position this as the first multi-institutional study of large HGB (>2cc) treated with SRS, which is a relevant and novel scope. However, the introduction could better justify the chosen >2cc threshold, as this cut-off seems somewhat arbitrary without robust prior evidence.
- The retrospective nature inherently limits conclusions, but selection bias is particularly relevant here. Only the largest tumor per patient was included, which might skew results toward worse baseline characteristics and outcomes. This decision should be justified.
- There is variability in radiosurgical equipment, planning, and follow-up protocols across 14 centers, which introduces heterogeneity. This needs more explicit acknowledgment and, ideally, sensitivity analyses.
- "Tumor control" is defined as regression or stability using ±20% volume change. This threshold is acceptable but should be referenced to established neuro-oncology standards or explained further.
- For OS and PFS, the definition of events (especially progression) should be clarified—was progression strictly radiographic, or were clinical criteria included?
- The conclusion that "SRS offers high tumor control rates with low morbidity" is reasonable, but the assertion that it is comparable to resection is speculative without a surgical control group.
- Authors should avoid implying equivalence between SRS and surgery; instead, they can state that SRS appears to be a viable alternative for selected large lesions.
- Adverse radiation effects are reported, but no standard grading system (e.g., CTCAE) is mentioned. Without grading, it's difficult to compare with other series.
- It is notable that a higher dose was associated with ARE, but the implications for dose selection should be more deeply discussed.

- The limitations section is present but underdeveloped. Additional points to include: (A) Lack of central imaging review; (B) Potential misclassification of progression vs. pseudoprogression; (C) Absence of quality-of-life or functional outcome measures; (D) Heterogeneity in prior treatments and timing of SRS.

Minor comments:

- Some sentences in the introduction are overly long and could be tightened for clarity.
- Ensure consistent terminology (e.g., “HGB” vs. “hemangioblastoma”).
- Abbreviations such as OS, PFS, and ARE should be defined at first mention in both the abstract and the main text.
